# Cortical Gray Matter Loss, Augmented Vulnerability to Speech-on-Speech Masking, and Delusion in People With Schizophrenia

**DOI:** 10.3389/fpsyt.2018.00287

**Published:** 2018-07-04

**Authors:** Chao Wu, Yingjun Zheng, Juanhua Li, Shenglin She, Hongjun Peng, Liang Li

**Affiliations:** ^1^Faculty of Psychology, Beijing Normal University, Beijing, China; ^2^Guangzhou Brain Hospital, Guangzhou Medical University, Guangzhou, China; ^3^School of Psychological and Cognitive Sciences, Beijing Key Laboratory of Behavior and Mental Health, Key Laboratory on Machine Perception, Ministry of Education, Peking University, Beijing, China; ^4^Beijing Institute for Brain Disorder, Capital Medical University, Beijing, China

**Keywords:** schizophrenia, speech perception, informational masking, delusion, gray-matter volume

## Abstract

People with schizophrenia exhibit impairments in target-speech recognition (TSR) against multiple-talker-induced informational speech masking. Up to date, the underlying neural mechanisms and its relationships with psychotic symptoms remain largely unknown. This study aimed to investigate whether the schizophrenia-associated TSR impairment contribute to certain psychotic symptoms by sharing underlying alternations in cortical gray-matter volume (GMV) with the psychotic symptoms. Participants with schizophrenia (*N* = 34) and their matched healthy controls (*N* = 29) were tested for TSR against a two-talker-speech masker. Psychotic symptoms of participants with schizophrenia were evaluated using the Positive and Negative Syndrome Scale. The regional GMV across various cortical regions was assessed using the voxel-based morphometry. The results of partial-correlation and mediation analyses showed that in participants with schizophrenia, the TSR was negatively correlated with the delusion severity, but positively with the GMV in the bilateral superior/middle temporal cortex, bilateral insular, left medial orbital frontal gyrus, left Rolandic operculum, left mid-cingulate cortex, left posterior fusiform, and left cerebellum. Moreover, the association between GMV and delusion was based on the mediating role played by the TSR performance. Thus, in people with schizophrenia, both delusions and the augmented vulnerability of TSR to informational masking are associated with each other and share the underlying cortical GMV reduction, suggesting that the origin of delusion in schizophrenia may be related to disorganized or limited informational processing (e.g., the incapability of adequately filtering information from multiple sources at the perceptual level). The TSR impairment can be a potential marker for predicting delusion severity.

## Introduction

Impairments in speech and thought processes have been considered as critical characteristics in people with schizophrenia (1–4). It has been suggested that investigation of the relationship between deficits of perceptual/cognitive processes and typical symptoms of schizophrenia is important for understanding the nature of this disorder (5–7). Since impaired inhibitory mechanisms at the neurobiological level in people with schizophrenia are associated with certain psychotic symptoms reflecting the incapability of adequately processing multiple inputs at the perceptual level (8–10), it is of importance to know whether deficits of perceptual/cognitive processes and certain symptoms in people with schizophrenia share the same or similar underlying neural substrates associated with impaired inhibitory processing.

Previous studies have shown that in adverse listening environments with multiple talkers, both people with first-episode schizophrenia and people with chronic schizophrenia exhibit a much larger difficulty in target-speech recognition (TSR) against informational speech-on-speech masking than their matched healthy controls (11–15), suggesting that the augmented vulnerability of TSR to irrelevant informational disrupting inputs can be used as a cognitive marker of schizophrenia. On the other hand, certain psychotic symptoms are also related to the reduced inhibition of irrelevant disrupting inputs (8–10). Up to date, whether the schizophrenia-related vulnerability of TSR to informational masking is associated with certain psychotic symptoms has not been reported in the literature. One research strategy for this issue is to investigate whether the vulnerability of TSR to informational masking and certain psychotic symptoms share the same or similar underlying neural substrates.

It has been known that the superior temporal gyrus (STG) is involved in not only processing of speech signals, but also informational masking of speech signals (14, 16–18). Also, the middle temporal gyrus (MTG) is involved in retrieval of auditory content-priming information during speech recognition against informational masking (14) and is a critical hub for sentence-level processing (19). Moreover, the inferior and middle frontal gyri, cingulate cortex, insular, and the cerebellum are all involved in processing of attended speech (12–15, 20, 21). Thus, structural/functional deficits of these cortical regions are likely related to impairments of TSR against informational masking in people with schizophrenia. As to psychotic symptoms in people with schizophrenia, the lateral temporal and the ventral frontal areas are associated with positive symptoms, especially delusions (22–24). Also, dysfunctions in the insular and cerebellum are related to delusions of control (i.e., self-produced actions are experienced as being externally produced) (25, 26). Thus, it is of importance and interest to know whether schizophrenia-related failures in filtering distracting speech signals and/or capturing relevant speech (which usually cause disorganization of speech-information processing) are related to both the enhanced vulnerability of TSR to informational masking and certain psychotic symptoms.

Previous studies on abnormal brain structures in schizophrenia have revealed the associations between impaired perceptual/cognitive processing (27–36) and clinical psychotic symptoms (28, 37, 38). Particularly, widespread reductions of gray-matter volume (GMV) in temporal and frontal areas are associated with dysfunctions of both cognitive control and response inhibition (35, 38). Besides, the GMV in the frontal, temporal, and parietal cortices in people with schizophrenia is negatively correlated with the severity of delusion and hallucination (27, 29, 38). Thus, encouraged by these previous reports, this study was to measure the abnormal GMV in people with schizophrenia (compared to that in healthy controls) and examine the hypothesis that certain schizophrenia-induced GMV alternations may underlie both impaired TSR and certain psychotic symptoms.

More specifically, this study aimed to examine whether the schizophrenia-related impairment of TSR against informational masking is associated with certain psychotic symptoms, and whether they share the common GMV alternations in certain brain regions. Accordingly, Spearman partial correlations and mediation analyses between GMV, TSR, and psychotic sympotms (with covariates incuding sex, age, educational years, ill-duration, and dosage of antipsychotics controlled) were conducted.

## Materials and methods

### Participants

Participants with schizophrenia were recruited from the Guangzhou Huiai Hospital. They were diagnosed according to the Structured Clinical Interview for DSM-IV (SCID-DSM-IV) (39). All the patient participants received antipsychotic medication during this study. Exclusion criteria: hearing loss, alcohol and/or drug abuse, nervous system disease, a treatment of the electroconvulsive therapy (ECT) within the past 6 months, a treatment of trihexyphenidyl hydrochloride with a dose of more than 6 mg/day, and/or an age either younger than 18 or older than 59 years (14, 21).

Healthy control participants were recruited from the communities near the Guangzhou Huiai Hospital. They were telephone interviewed first and then were screened with the SCID-DSM-IV as used for patient participants during clinical interview. None of the healthy participants had a history of Axis I psychiatric disorder as defined by the DSM-IV. Both patient participants and their demographically-matched healthy controls underwent both the behavioral testing and the structural MRI scanning.

All participants (34 participants with schizophrenia and 29 healthy controls) were right-handed, and their first language was mandarin Chinese. They did not show any pure-tone hearing impairments for each ear at the frequencies of 125, 256, 512, 1,024, and 2,048 Hz. Some of them also participated in our previous study (21). Both the participants (including patient participants and healthy controls) and the guardians of the patient participants gave their written informed consent for participation in this study. The procedures of this study were approved by the Independent Ethics Committee (IEC) of the Guangzhou Huiai Hospital. The investigation was carried out in accordance with the latest version of the Declaration of Helsinki.

### Stimuli

Target-speech stimuli were Chinese nonsense sentences, which are syntactically correct but not semantically meaningful (providing none contextual support for recognizing a keyword) (14, 21, 40). For example, the English translation of a Chinese nonsense sentence is “One appreciation could retire his ocean” (keywords are underlined). Each of the Chinese sentences has 12 syllables (also 12 characters) including three keywords with two syllables for each.

The masking-speech stimulus was a 47-s loop of digitally combined continuous recordings for Chinese nonsense sentences spoken by two different young female talkers. In a trial, the duration of each masking-speech stimuli was 1,000 ms longer than that of its target speech stimulus.

**Figure 1:**
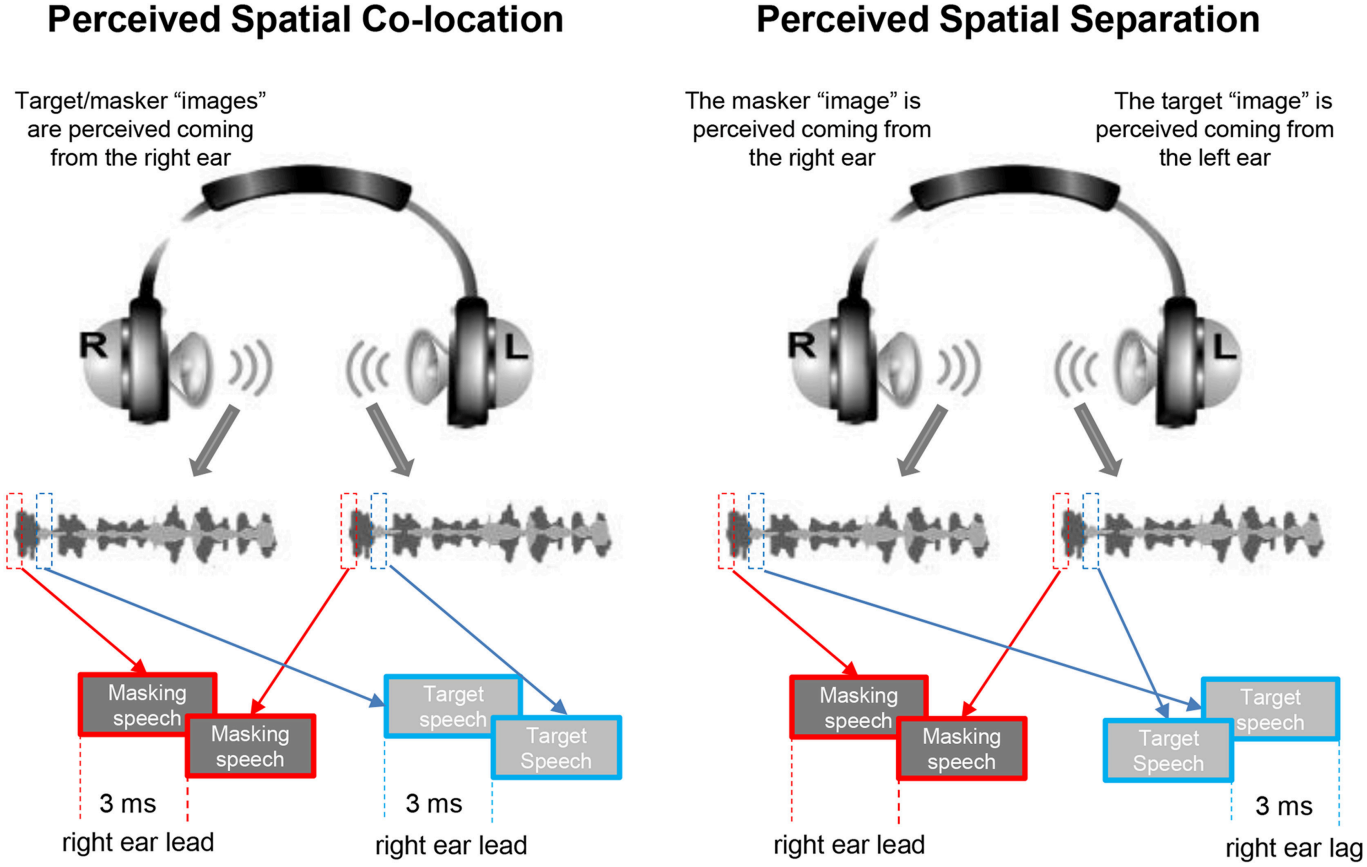
Based on the auditory precedence-effect paradigm and the head-related transfer function (HRTF), the target speech and masking speech were simulated as being presented by each of the two spatially separated “loudspeakers” in the frontal field with the inter-source interval of 3 ms. Under the perceived spatial co-location (PSC) condition (left panel), both the onset of the target sound and that of the masker sound presented from the right headphone led those from the left headphone by 3 ms, leading to a perceptually fused target sound “image” and a perceptually fused masker “image” as coming from the same right location. On the other hand, under the perceived spatial separation (PSS) condition (right panel), when the onset of the target sound presented from the left headphone led that from the right headphone by 3 ms, and the onset of the masker sound presented from the left headphone lagged behind that from the right headphone by 3 ms, due to the precedence effect, the perceptually fused target image was perceived as coming from the left location and the perceptually fused masker image was perceived as coming from the right location.

An auditory-precedence-effect (40) induced perceived spatial separation (PSS) was introduced as the unmasking cue (for improving TSR) against the precedence-effect-induced perceived spatial co-location (PSC) condition (10, 20). Specifically, the speech signals were digitally processed with head-related transfer functions (HRTFs) to generate virtual sound images that appeared to occur under free-field listening conditions. The speech signals for a single voice were filtered with the HRTFs to simulate source locations at both 90° left and 90° right to the listener in the azimuth (21, 41). Under the PSS condition, due to the auditory precedence effect, the image of target speech and that of masking speech were perceived as coming from different loudspeaker positions (the leading ear for target speech was different from that for masking speech), causing that the target speech was released from informational masking, because the selective attention to the target speech was facilitated. However, under the perceived spatial co-location (PSC) condition, also due to the precedence effect, the image of target speech and that of masking speech were perceived as coming from the same loudspeaker positions (the leading ear for target speech was the same as that for masking speech), causing the maximal informational masking (Figure 1). Note that shifts between the PSS condition and the PSC condition altered neither the signal-to-masker ratio (SMR) nor the perceived compactness of the sound images.

### Speech-recognition task

The participant was seated at the center of a quiet room in the hospital. Acoustic signals, calibrated by a sound-level meter (AUDit and System 824, Larson Davis, USA), were delivered from a notebook computer to earphones (Model HAD 200) and bilaterally presented to the participant at the sound pressure level (SPL) of 60 dBA. The SPLs of the masker were adjusted to produce four SMRs: −8, −4, 0, and 4 dB.

For each participant, there were two within-subject variables: (1) spatial condition (PSS, PSC), and (2) SMR (−8, −4, 0, and 4 dB). There were 8 testing conditions in total with 12 trials (also 12 target sentences) in each condition. The presentation order for the 4 SMRs was arranged randomly for each of the stimulation conditions for each participant. The presentation order for these spatial conditions was pseudorandomized across participants for each group. In a single trial, the stimuli were presented following the participant pressed the “Enter” key on a computer keyboard. After the masker/target co-presentation was finished, the participant was instructed to loudly repeat the whole target sentence as best as she/he could. Responses for each of the two syllables for the target keywords of the target sentence were recorded (20).

### Assessments of the target-speech recognition

A logistic psychometric function,

$$\begin{array}{l} \text{y}=1/\left[1+{\text{e}{}^{-\text{σ}}}^{\left(x-\mu \right)}\right] \end{array}$$

was fit to each individual participant's data, using the Levenberg-Marquardt method (42), where y is the probability of correct recognition of the last (third) target keywords, x is the SMR corresponding to y, μ (the threshold) is the SMR corresponding to 50% correct on the psychometric function, and σ determines the slope of the psychometric function. The lower the μ is, the better the speech-recognition performance is. Here, the TSR performance for a participant was defined by the μ-value averaged across the PSS listening condition and the PSC listening condition. The unmasking effect induced by PSS was defined by the difference in μ (absolute value) between the PSS listening condition and the PSC listening condition (|μ_PSS_ –μ_PSC_|).

### Measures of psychotic symptoms

The locally validated (Chinese Mandarin) version of the Positive and Negative Syndrome Scale (CMV-PANSS) (43–45) was conducted for each patient participant on the day they took the speech recognition test. CMV-PANSS total and group-mean scores for the five dimensions of psychotic symptoms (positive, negative, disorganized cognition, excited/activation, and emotional distress) (44, 46) were calculated.

### Structural MRI data acquisition

A 3.0-Tesla MRI system (Achieva Scanner; Philips, Veenpluis, Netherlands) was used to acquire the high-resolution T1-weighted structural volumetric sequence [256 × 256 × 188 matrix with a spatial resolution of 1 × 1 × 1 mm^3^, repetition time (TR): 8.2 ms; echo time: 3.8 ms; flip angle: 7°] covering the whole brain. The scanning duration was 8 min.

### Statistical analyses

#### VBM-DARTEL analyses

Voxel-based morphometry (VBM) analyses were performed using SPM12 (http://www.fil.ion.ucl.ac.uk/spm/) and MATLAB 7.10.0. The pre-processing included the following steps: (1) Images were segmented into gray matter (GM), white matter (WM), and cerebrospinal fluid (CSF) using the segmentation model with default parameters (47). The outputs of the first step were a series of rigidly aligned tissue class images. (2) To estimate the non-linear deformations that best aligned the tissue class images all together, GM population templates were generated by the diffeomorphic anatomical registration using the exponentiated Lie algebra (DARTEL) technique (48). (3) To create the Jacobian scaled warped tissue class images, both an affine transform of the population average (DARTEL Template space) templates to the Montreal Neurological Institute MNI space and smoothness with an 8 mm full width at half maximum Gaussian kernel were performed.

After the spatial pre-processing, the smoothed, modulated, normalized GM volumes were entered into a two-sample two-tailed *t*-test between the health controls and patients with schizophrenia to create the group-mean map of abnormal gray matter. The threshold for the *T*-value map was set at *p* < 0.05 (cluster-wise FWE corrected). Subsequently, a region of interest (ROI) was defined as a sphere with 5-mm radius centered at the peak voxel (Marsbar, http://marsbar.sourceforge.net) of each cluster in the map of abnormal gray matter. Then, the mean GMV within each of the identified ROIs for each participant was calculated and extracted using FMRIB Software (https://fsl.fmrib.ox.ac.uk/fsl/fslwiki/).

#### Partial-correlation and mediation analyses

Analyses were performed using SPSS 20.0 and R 3.2.3. Independent sample *t*-test or Pearson's chi-square test was conducted to compare the characters between groups. Partial Spearman correlation analyses were used to test the relationships between GMVs, behavioral (TSR or unmasking effect) scores, and psychotic symptoms in participants with schizophrenia, with potential covariates (age, sex, educational years, ill-duration, and dosage of antipsychotics) controlled. The Benjamini-Hochberg standard false discovery rate (FDR) method was used for correcting *p-*values for multiple comparisons.

Mediation analyses were used to investigate the indirect effect of TSR on the link between the decreased GMV and the psychotic symptoms in participants with schizophrenia, with covariates controlled. The bootstrapping method was used to test the sampling distributions (5,000 times resampling) of the indirect (mediation) effects. The bootstrap confidence interval (CI) for the indirect effect through the mediator was derived by sorting the 5,000 values from low to high. Values defining the lower and upper 100 (α/2) % (α = 0.05; the desired nominal Type I error rate) of the distribution were found and taken as the lower and upper limits of the 100 (1-α) % CI for the indirect effect (49).

## Results

### Characteristics and behavioral performance of participants

Participants with schizophrenia (*n* = 34, age = 31.3 ± 8.6 years) and their healthy controls (*n* = 29, age = 29.4 ± 7.4 years) showed no significant differences in age, sex, and educational years. Averagely, for participants with schizophrenia, the chlorpromazine equivalent, which was calculated using conversion factors described by Woods (50), was 508 ± 199 mg/day (Table 1).

**Table 1:** Demographic, clinical characteristics, and behavioral performance in participants with schizophrenia and healthy controls.

**Characteristic**	**SCH (*n* = 34)**	**HC (*n* = 29)**	**t/χ^2a^**	***p***
	**Mean (SD)**	**Mean (SD)**		
Age (years)	31.32 (8.59)	29.4 (7.61)	1.85	0.158
Male% (n)	58.8 (20)	43.3 (13)	1.88	0.599
Education (years)	13.23 (3.17)	14.3 (2.22)	1.04	0.339
Ill-duration (years)	6.87 (6.00)	NA	NA	NA
CMV-PANSS total	54.00 (5.79)	NA	NA	NA
Positive	15.47 (4.60)	NA	NA	NA
Negative	12.20 (3.93)	NA	NA	NA
Cognition	12.79 (2.64)	NA	NA	NA
Emotion	6.47 (2.21)	NA	NA	NA
Excitation/Aggression	7.68 (1.20)	NA	NA	NA
Speech Recognition (μ)	−3.46 (1.99)	−6.73 (1.96)	6.56	< 0.001
Priming Effect (μ)	1.83 (3.06)	3.82 (3.01)	2.60	0.012
Chlorpromazine ED (mg)	508.01 (198.68)	NA	NA	NA
Typical (number)	15	NA	NA	NA
Atypical (number)	29	NA	NA	NA
Typical/atypical (number)*	9	NA	NA	NA

SD, standard deviation; ED, equivalent doses; CMV-PANSS, positive and negative syndrome scale; NA, not applicable. *Note that 9 patients received 2 different antipsychotic medications.

a *Analyses were conducted between the two patient groups by t-tests for normally distributed variables and χ^2^-tests for categorical variables*.

**Figure 2:**
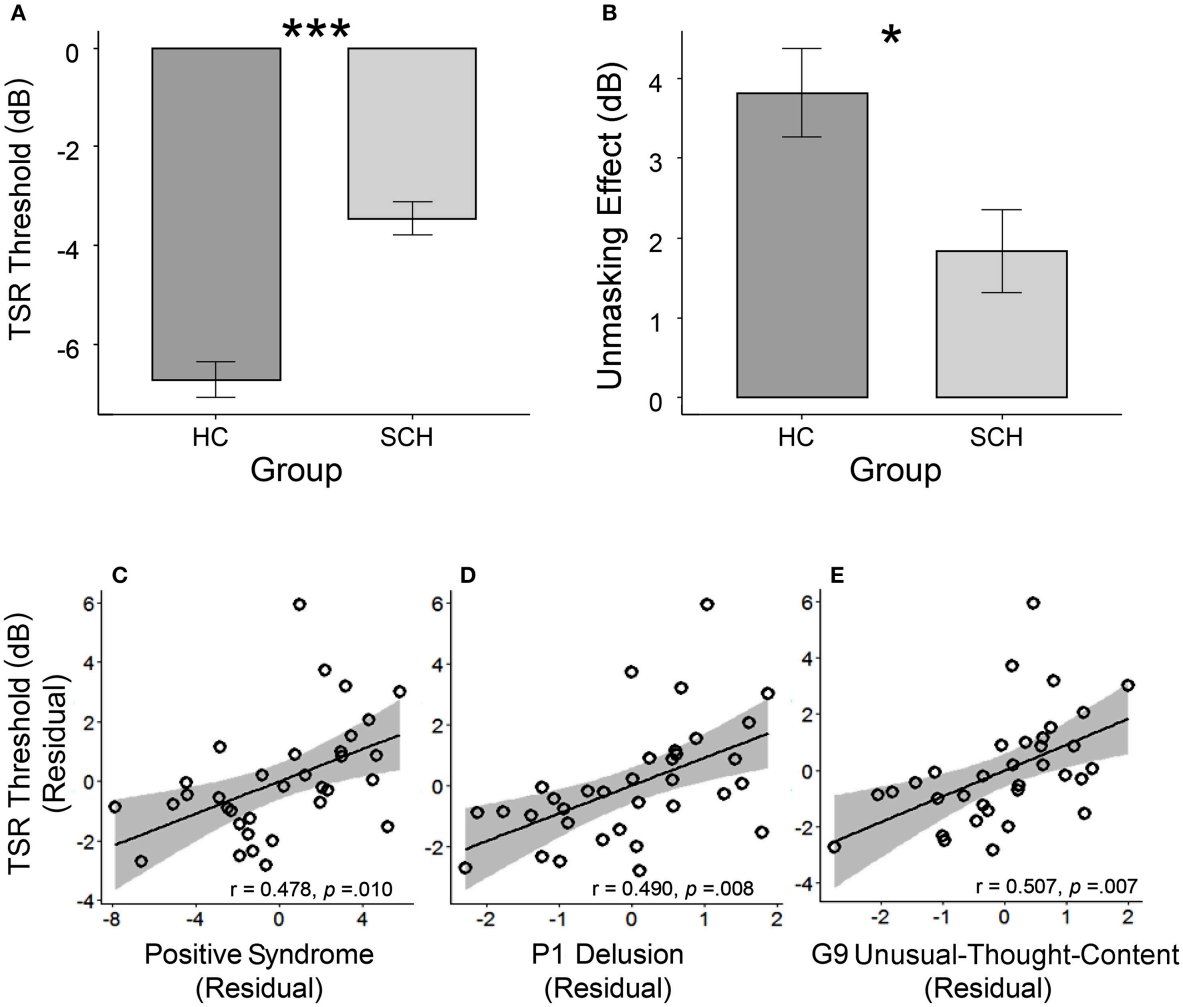
**(A)** The group-mean target-speech-recognition threshold (μ) was significantly higher (the higher the μ is, the poorer the speech recognition is) in the group with schizophrenia (SCH) than that in the group of healthy controls (HC). **(B)** The unmasking effect (Δμ) induced by the perceived spatial separation in the SCH group was significantly smaller than that in the HC group. In the SCH group, the bottom panels illustrate the partial regression plots for the significant correlations between the TSR threshold and the CMV-PANSS positive syndrome **(C)**, CMV-PANSS-P1 (delusion) **(D)**, and CMV-PANSS-G9 (unusual thought content) **(E)** with the statistical controls for age, sex, education, ill duration, medication dosage, and CMV-PANSS-total. *p < 0.05; ***p < 0.001.

Participants with schizophrenia had poorer TSR (higher μ-value) than their healthy controls (mean μ was −3.5 dB for the patient participants and −6.7 dB for the healthy controls; *t* = 6.56, *p* < 0.001; Cohen's *d* = 1.66, and 95% CI ranged between 1.07 and 2.24) (Table 1 and Figure 2A). Patient participants also had lower release of target speech from informational masking than healthy controls (mean Δμ was 1.8 dB for patient participants and 3.8 dB for healthy controls; *t* = 2.60, *p* = 0.012; Cohen's *d* = 0.66, and 95% CI ranged between 0.14 and 1.18) (Table 1 and Figure 2B).

### Partial correlation between speech recognition threshold and psychotic symptoms

For participants with schizophrenia, significant Spearman partial correlation was found between the positive syndrome and the TSR threshold (μ) (*r* = 0.478, *p* = 0.010, FDR-corrected *p* = 0.044) (Figure 2C). No significant correlation was found between TSR threshold and other symptom dimensions of CMV-PANSS (Figure S1). Further inspection on each item of CMV-PANSS positive syndromes showed that both the P1 (delusion) score (*r* = 0.490, *p* = 0.008, FDR-corrected *p* = 0.015) and the G9 (unusual thought content) score (*r* = 0.501, *p* = 0.007, FDR-corrected *p* = 0.015) contributed to the positive correlation between CMV-PANSS positive syndrome and the μ-value (Figures 2D,E). Thus, for participants with schizophrenia, the more severe the positive syndromes (especially for delusion and unusual thought content) were, the worse (higher) the TSR threshold μ (against informational masking) was. Also, no significant correlation was found between the unmasking effects and the psychotic symptoms for participants with schizophrenia.

### Mediating effects of target-speech recognition on the association between decreased gray-matter volume and delusion severity

**Figure 3:**
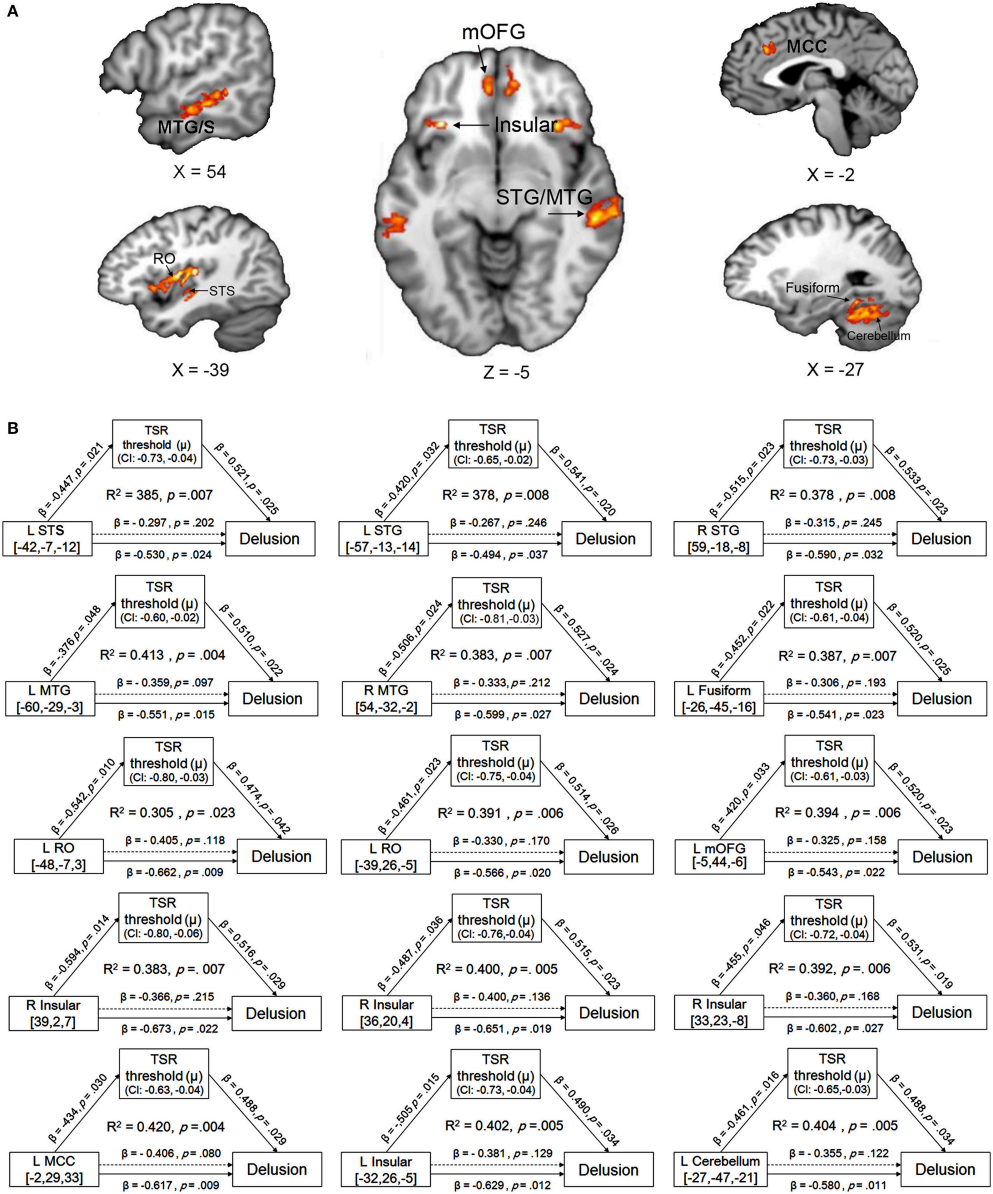
**(A)** Brain regions with reduced gray-matter volume (GMV) in participants with schizophrenia, compared to their demographically-matched healthy controls. A cluster-defining threshold (CDT) (*p* = 0.001; *T* = 3.23) and a cluster based FWE-corrected threshold (*p* = 0.05) was used. The map was overlaid on the template from the Mango software (http://rii.uthscsa.edu/mango//index.html). **(B)** The mediating effects of the impaired target-speech recognition (TSR) on the relationships between the decreased GMV and the delusion symptom in participants with schizophrenia. Adjusted *R*^*2*^, standardized regression coefficients, *p*-values and bias-corrected confidence interval (95% CI) for the mediation effect were shown. Arrows with solid lines indicate that the effects were significant, and arrows with dashed lines indicate that the effects were not significant. MCC, mid-cingulate cortex; mOFG, medial orbital frontal gyrus; RO, Rolandic operculum; STG, superior temporal gyrus; STS, superior temporal sulcus, MTG, middle temporal gyrus; MTS, middle temporal sulcus.

Compared to their demographically-matched healthy controls, participants with schizophrenia showed a reduction of GMV in the following 18 brain regions, which were treated as the regions of interest (ROIs) in this study: bilateral superior temporal gyrus/sulcus (STG/STS), bilateral middle temporal gyrus/sulcus (MTG/MTS), left posterior fusiform, bilateral insular, bilateral medial orbital frontal gyrus (mOFG), left Rolandic operculum (RO), left mid-cingulate cortex (MCC), left posterior fusiform and left cerebellum (Figure 3A and Figure S2 in the online Supplementary Materials) (with the threshold set at *p* < 0.05 (FWE corrected) on the cluster level with a cluster-defining threshold (CDT) of *p* = 0.001 (*T* = 3.23), uncorrected).

The GMV of each of the 18 brain ROIs was entered into correlation and mediation analyses. The Spearman partial correlation coefficients between the GMV and the TSR threshold, and those between the GMV and the two CMV-PANSS positive syndromes (CMV-PANSS-P1, CMV-PANSS-G9) are shown in Table 2. In participants with schizophrenia, the correlation between the GMV and the TSR threshold was significant for 15 of the 18 ROIs (corrected *p* < 0.05). However, in healthy participants no significant correlations between the TSR threshold and the GMV were found for both these 18 ROIs and other brain regions (with age, sex, and educational years controlled).

**Table 2:** Coefficients of spearman partial correlation between gray matter volume of rois, target-speech-recognition threshold (μ), and P1/G9 Score of CMV-PANSS in participants with schizophrenia.

**Brain region**	**MNI coordinate**	**Speech recognition**			**P1-delusion**			**G9-Unusual-thought-content**		
		***r***	***p***	***p*^corr^**	***r***	***p***	***p*^corr^**	***r***	***p***	***p*^corr^**
L mOFG	[−5,44,−6]	−0.453*	0.016	**0.039**	−0.472*	0.011	**0.032**	−0.400	0.035	0.089
L P Fusiform	[−26,−45,−12]	−0.411*	0.030	**0.042**	−0.484*	0.009	**0.032**	−0.391	0.039	0.089
L Cerebellum	[−36,−50,−27]	−0.454*	0.015	**0.039**	−0.468*	0.012	**0.032**	−0.397	0.036	0.089
L Cerebellum	[−27,−47,−2]	−0.488*	0.008	**0.039**	−0.550*	0.002	**0.032**	−0.536	0.003	0.054
L Insular	[−32,26,−5]	−0.479*	0.010	**0.039**	−0.485*	0.009	**0.032**	−0.428	0.023	0.089
L MCC	[−2,29,33]	−0.433*	0.021	**0.039**	−0.425*	0.024	**0.032**	−0.355	0.064	0.089
L MTG	[−60,−29,−3]	−0.429*	0.023	**0.039**	−0.437*	0.020	**0.032**	−0.382	0.045	0.089
L OR	[−39,−18,12]	−0.406*	0.032	**0.042**	−0.396*	0.037	**0.039**	−0.319	0.098	0.098
L OR	[−48,−7,3]	−0.403*	0.033	**0.042**	−0.432*	0.022	**0.032**	−0.360	0.060	0.089
L STG	[−42,−7,−12]	−0.377	0.048	0.051	−0.457*	0.015	**0.032**	−0.367	0.055	0.089
L STG	[−57,−3,−14]	−0.361	0.059	0.059	−0.487*	0.009	**0.032**	−0.346	0.072	0.089
R mOFG	[1,36,−14]	−0.431*	0.022	**0.039**	−0.428*	0.023	**0.032**	−0.381	0.045	0.089
R Insular	[33,23,−8]	−0.380	0.046	0.051	−0.417*	0.027	**0.032**	−0.325	0.091	0.096
R Insular	(4, 20, 34)	−0.399*	0.036	**0.043**	−0.441*	0.019	**0.032**	−0.343	0.074	0.089
R Insular	(2, 7, 39)	−0.459*	0.014	**0.039**	−0.431*	0.022	**0.032**	−0.352	0.066	0.089
R MTG	[54,−32,−2]	−0.426*	0.024	**0.039**	−0.417*	0.027	**0.032**	−0.388	0.041	0.089
R MTG	[65,−24,−6]	−0.428*	0.023	**0.039**	−0.406*	0.032	**0.036**	−0.362	0.058	0.089
R STG	[59,−15,−8]	−0.468*	0.012	**0.039**	−0.336	0.080	0.080	−0.334	0.083	0.093

mOFG, medial orbital frontal gyrus; MCC, mid-cingulate cortex; MTG, middle temporal gyrus; OR, olandic operculum; STG, superior temporal gyrus; L, left; R, right; P, posterior. The p-value was obtained controlling for age, sex, education years, ill-duration, medication dosage of antipsychotics and total score of CMV-PANSS. The p^corr^ was corrected by the Benjamini-Hochberg standard false discovery rate (FDR) method. The bold emphases indicate significant correlations corrected by the FDR method.

* *p < 0.05*.

Also, the correlation between GMV and CMV-PANSS-P1 (delusion) was significant for 17 brain regions of the 18 ROIs (corrected *p* < 0.05; Table 2). However, no significant correlation was found between GMV and CMV-PANSS-G9 for each of the ROIs (no survivors for multiple comparisons).

The mediation models of the TSR threshold (μ-value) on the relationships between GMV and delusion (CMV-PANSS-P1) severity were significant for all the 15 ROIs as indicated in Figure 3B (*R*^2^ ranged from 0.305 to 0.420, *p*-values ranged from 0.023 to 0.004). The direct effects of GMV on delusion severity (CMV-PANSS-P1) were also significant for all the 15 ROIs (standardized β ranged from −0.494 to −0.673, *p*-values ranged from 0.037 to 0.009), when age, sex, educational years, ill-duration, medication dosage, and CMV-PANSS total were controlled. The GMV for each of the 15 ROIs was significantly associated with the TSR threshold (μ-value) when the covariates were controlled (standardized β ranged from −0.376 to −0.594, *p* values ranged from 0.048 to 0.014), and the TSR threshold was significantly associated with the delusion severity for each of the 15 models (standardized β ranged from 0.474 to 0.541, *p*-values ranged from 0.042 to 0.019).

Moreover, after the adjustment by the mediator of the TSR and other covariates, the correlation between GMV and delusion became not significant (standardized β ranged from −0.306 to −0.406, *p-*values ranged from 0.215 to 0.080). Bootstrapping sampling (*n* = 5,000) confirmed that the effect of GMV on the delusion severity through the TSR was significant for each of the 15 models (both the lower limit and the upper limit of the 95% bootstrap confidence interval were below zero) (Figure 3B). Thus, the TSR threshold (against informational speech masking) significantly mediated the association between the delusion severity and the GMV changes in the frontal cortices (left mOFG, and left RO), temporal cortices (bilateral STG, bilateral MTG, and left posterior fusiform), bilateral insular, left MCC, and left cerebellum.

## Discussion

This study, for the first time, investigated the associations between GMV, TSR against informational masking, and psychotic symptoms in people with schizophrenia. The results showed that the delusion severity and the TSR threshold against informational masking were correlated to each other and shared the common reduction of GMV in the following brain regions: the bilateral STG/STS, bilateral MTG, left posterior fusiform, left mOFG, bilateral RO, left MCC, and left cerebellum. More importantly, the TSR against informational masking played a mediating role in the association between the GMV and the delusion symptom severity, suggesting that the schizophrenia-enhanced vulnerability of TSR to informational masking contributes to the delusion severity due to certain brain-structure impairments.

### Speech recognition against informational masking is negatively correlated with delusion severity and unusual thought content

This study for the first time reveals that in people with schizophrenia the ability in TSR against informational speech masking is negatively correlated with the severity of the positive syndromes, including the delusion and unusual-thought symptoms on the CMV-PANSS. In other words, if a patient with schizophrenia has a higher threshold μ for recognizing target speech (poorer speech-recognition performance) against informational masking, this patient has a higher severity of the delusion and unusual thought content. Thus, the speech-recognition impairment under informational masking conditions is useful for predicting the delusion and unusual-thought severity in people with schizophrenia.

### Target-speech recognition deficits and delusion share certain underlying pathophysiological mechanisms

This study discovers that the delusion symptom severity is associated with the reduced TSR against informational masking, and the TSR mediates the association between the delusion symptom and the reduced GMV in somce brain regions known to be normally involved in not only processing of masked speech (11–18, 20, 21) but also cognitive controls (51, 52). Moreover, abnormal functions of these brain regions are related to the risk of the delusion symptom (4, 27, 38, 53). Thus, it can be suggested that the association between the GMV loss in these brain areas and the impairment of TSR against informational speech masking may reflect the deficiency in speech “gating” (i.e., the failure in either filtering out distracting speech streams or capturing relevant speech streams) in people with schizophrenia. It should be noted that with mediation analyses, the directional causes among GMV, CMV-PANSS, and TSR can be only speculated.

In this study, beyond the lateral superior/middle temporal and the ventral frontal areas, direct correlations between delusion symptom, TSR (against informational masking), and GMV were also observed for the left anterior MCC and left Rolandic Operculum in participants with schizophrenia. It has been suggested that functional impairments of these two brain regions in the salience network are linked to delusional thoughts in schizophrenia. In particular, the anterior MCC is a critical node of the salience network (54, 55) and involved in modulating the salience of inner or outer speech information in schizophrenia. Also, the left Rolandic operculum is involved in syntactic encoding during speech production (56) and sentence-level speech prosody processing (57), implying that biased processing of speech information induced by deficits in the Rolandic Operculum may be related to thought disorder in schizophrenia.

The reduction in GMV, which is correlated with both the impairment of TSR and the occurance of delusion, may be associated with the schizophrenia-related dysfunctions of source monitoring. It has been reported that the temporal and the middle/inferior frontal gyri are involved in reality monitoring (58), and hypo-activation of the neural network comprised of the thalamus and frontotemporal regions may underlie impaired speech monitoring in schizophrenia (59).

Furthermore, it is of interest to know whether the GMV loss-induced deficits of “gating” or “monitoring” underlying both the delusion symptom and the speech-recognition impairment are related to certain alternations of neurotransmissions in the frontal and temporal association cortices. For example, the Xie et al. study (60) has shown that the dopamine D4 receptor (DRD4) gene, which is primarily expressed in the middle and inferior frontal gyri (61), is linked to informational masking of speech (i.e., TSR against masking speeches) (60). Moreover, in people with schizophrenia, both the loss of pyramidal cells in the cortex (especially in temporal and frontal regions) (62, 63) and the functional declines of dopaminergic and glutamatergic signaling in the frontal and/or auditory association cortices contribute to the delusion formation (4, 53, 64–67). Thus, in the future it is important to investigate whether the impairments of “gating” or “monitoring” functions of the frontal and temporal association cortices in people with schizophrenia are specifically associated with dopaminergic transmission deficits, leading to both the impairment of speech recognition against informational masking and the occurrence of delusion.

## Conclusions

This study for the first time reveals that speech recognition against informational masking is negatively correlated with the severity of delusion and unusual-thought content in people with schizophrenia, suggesting that the speech-recognition impairment under informational masking conditions is useful for predicting the severity of the positive symptoms. Moreover, both the severity of delusion and the impairment of speech recognition against informational masking share the common GMV loss in the brain regions that are associated with speech filtering and source monitoring. These findings support the view that delusion is related to abnormal processing of informational “filtering” induced by GMV reduction, thereby providing a novel perspective for understanding the mechanisms underlying schizophrenia.

## Author contributions

LL, CW, and YZ designed the study, and wrote the first draft of the manuscript. CW carried out all statistical analyses. CW, YZ, JL, SS, and HP conducted data collection. All authors contributed to and approved the final version of the manuscript.

### Conflict of interest statement

The authors declare that the research was conducted in the absence of any commercial or financial relationships that could be construed as a potential conflict of interest.
