# Supplementary material for: Cortical Gray Matter Loss, Augmented Vulnerability to Speech-on-Speech Masking, and Delusion in People With Schizophrenia
Source: Front Psychiatry. 2018 Jul 4;9:287. doi: 10.3389/fpsyt.2018.00287 (PMC6040158; doi:10.3389/fpsyt.2018.00287)
Supplement: Supplementary file 1 [file Presentation_1.pdf]

SUPPLEMENTARY MATERIALS

Cortical Gray Matter Loss, Augmented Vulnerability to Speech-on-Speech Masking, and Delusion in People with Schizophrenia

(**Running Title**: Delusion and Informational Masking of Speech)

Chao Wu[a], Yingjun Zheng[b], Juanhua Li[b], Shenglin She[b], Hongjun Peng[b], Liang Li[c,d]

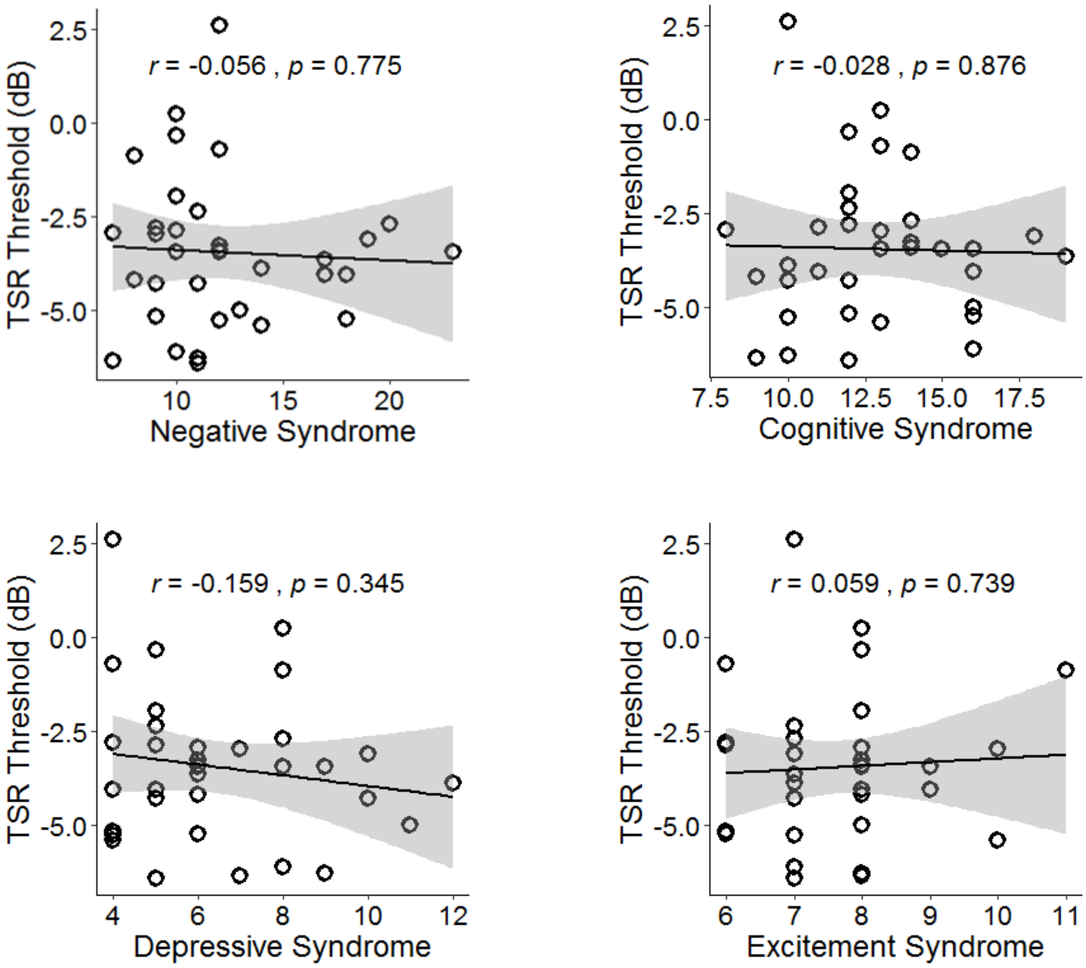

Figure S1. There were no significant correlations between the TSR threshold and the negative syndrome, the cognitive syndrome, the depressive/emotion syndrome, and the excitement syndrome of the CMV-PANSS.

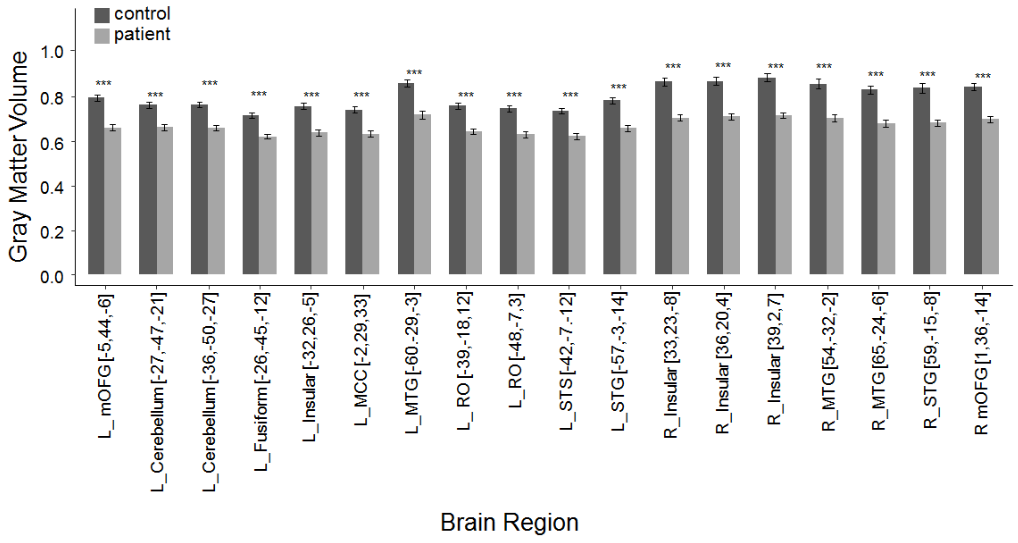

Figure S2. Bar plot for group comparison in gray matter volume. MCC, mid-cingulate cortex; mOFG, medial orbital frontal gyrus; RO, rolandic operculum; STG, superior temporal gyrus; STS, superior temporal sulcus; MTG, middle temporal gyrus.
